# Impact of Three Consecutive Days of Endurance Training Under Hypoxia on Muscle Damage and Inflammatory Responses

**DOI:** 10.3389/fspor.2021.663095

**Published:** 2021-04-15

**Authors:** Daichi Sumi, Keiichi Yamaguchi, Kazushige Goto

**Affiliations:** ^1^Research Center for Urban Health and Sports, Osaka City University, Osaka, Japan; ^2^Research Fellow of Japan Society for the Promotion of Science, Tokyo, Japan; ^3^Research Organization of Science and Technology, Ritsumeikan University, Kusatsu, Japan; ^4^Graduate School of Sports and Health Science, Ritsumeikan University, Kusatsu, Japan

**Keywords:** hypoxia, endurance training, muscle damage, inflammation, fatigue

## Abstract

**Purpose:** The purpose of this study was to determine the effect of 3 consecutive days of endurance training under hypoxia on muscle damage, inflammation, and performance responses.

**Methods:** Nine active healthy males completed two trials in different periods, consisting of either 3 consecutive days of endurance training under hypoxia [fraction of inspired oxygen (Fio_2_): 14.5%, HYP] or normoxia (Fio_2_: 20.9%, NOR). They performed daily 90-min sessions of endurance training consisting of high-intensity endurance interval pedaling [10 × 4-min pedaling at 80% of maximal oxygen uptake ($\dot{\text{V}}{\text{o}}_{\text{2max}}$) with 2 min of active rest at 30% of $\dot{\text{V}}{\text{o}}_{\text{2max}}$] followed by 30-min continuous pedaling at 60% of $\dot{\text{V}}{\text{o}}_{\text{2max}}$ during 3 consecutive days (days 1–3). Venous blood sample, muscular performance of lower limb, and score of subjective feelings were determined every morning (days 1–4) to evaluate muscle damage and inflammation. On day 4, subjects performed an incremental exercise test (IET) to evaluate the performance response.

**Results:** Pedaling workload during daily endurance training was significantly lower in the HYP trial (interval exercise: 166 ± 4 W) than in the NOR trial (194 ± 8 W; *P* < 0.0001). Serum creatine kinase (CK) and high-sensitivity C-reactive protein (hsCRP) concentrations did not significantly change during days 1–4 in either trial. Maximal voluntary contraction (MVC) of knee extension (*P* < 0.0001) and drop jump (DJ) index (*P* = 0.004) were significantly decreased with training in both trials, with no significant difference between trials. The muscle soreness and fatigue scores significantly increased in both trials (*P* < 0.0001). However, the HYP trial showed a significantly lower score of fatigue on day 4 compared with the NOR trial (*P* = 0.004). Maximal aerobic power output during IET on day 4 did not significantly differ between trials.

**Conclusion:** Three consecutive days of endurance training under hypoxia induced comparable levels of muscle damage, inflammation, and performance responses compared with the same training under normoxia.

## Introduction

The use of training under hypoxia (hypoxic training) is widely accepted among endurance athletes to improve endurance capacity, and considerable evidence supports the efficacy of this training procedure (Dufour et al., 2006; Czuba et al., 2011, 2017). Moreover, hypoxic training improves metabolic risk markers (e.g., glycemic regulation) in both healthy and obese individuals (Haufe et al., 2008; Mackenzie et al., 2012; De Groote et al., 2018). Many previous studies have investigated the effects of hypoxic training on exercise capacity or health status, whereas the effects on muscle damage and inflammatory responses have not been well-established. Strenuous exercise transiently reduces muscle strength and power and increases delayed-onset muscle soreness, muscle swelling, and serum levels of muscle damage markers [e.g., creatine kinase (CK)] and inflammatory cytokines [e.g., high-sensitivity C-reactive protein (hsCRP)] during post-exercise (Goto and Morishima, 2014; Mizuno et al., 2016; Peake et al., 2017). Exercise-induced muscle damage and inflammatory responses may play a role in promoting muscle mass and muscle regeneration (Allen et al., 1999; Britto et al., 2020), whereas excessive responses during training period would suppress training adaptations (Smith, 2003). Thus, information regarding muscle damage and inflammatory responses during the training period would help prevent overtraining syndrome or decrease the risk of injury (e.g., muscle strain) in working muscle (Smith, 2003; Meeusen et al., 2013).

Some of the previous studies demonstrated the effect of a single session of exercise under hypoxia on muscle damage and inflammatory response (Sumi et al., 2018; Britto et al., 2020; Hill et al., 2020). In our previous study, we compared the acute responses of muscle damage and inflammation following a single session of endurance exercise between hypoxia and normoxia. Endurance exercise-induced serum myoglobin elevation (an indirect muscle damage marker) during the post-exercise period (0–2 h) was significantly lower in hypoxia than in normoxia, when the exercise intensity was relatively matched [% of maximal oxygen uptake ($\dot{\text{V}}{\text{o}}_{\text{2max}}$)]. Moreover, reduction of the endurance capacity (evaluated by time to exhaustion) 2 h after completing endurance exercise was significantly lower in hypoxia than in normoxia. In contrast, exercise-induced interleukin 6 (IL-6) elevation (inflammatory cytokine) did not differ between hypoxia and normoxia (Sumi et al., 2018). These results suggest that endurance exercise under hypoxia causes less exercise-induced muscle damage and reduction of endurance capacity compared with the same exercise under normoxia using equivalent relative exercise intensity. However, the above study used a single session of endurance exercise. Currently, the cumulative effect of endurance training under hypoxia on muscle damage and inflammatory responses remains unclear.

Therefore, the purpose of this study was to determine the effect of 3 consecutive days of endurance training under hypoxia on muscle damage and inflammatory responses compared with the same training under normoxia. We hypothesized that endurance training under hypoxia causes less muscle damage and inflammatory responses compared with the same training under normoxia.

## Materials and Methods

### Subjects

Nine healthy, non-smoking male subjects participated in this study. The mean and SD for age, height, and body mass were 21.6 ± 0.8 years, 168.1 ± 2.1 cm, and 61.5 ± 2.1 kg, respectively. All subjects were physically active and involved in recreational resistance exercise or endurance exercise at the start of the experiment. They were born and living at sea level. All subjects were informed of the experimental procedures and possible risks involved in this study, and informed consent was subsequently obtained. This study was approved by the ethics committee for Human Experiments at Ritsumeikan University (BKC-IRB-2018-093) and was conducted in accordance with the Declaration of Helsinki.

### Experimental Design

This was a randomized, single-blind crossover study. All subjects completed two trials on different weeks, consisting of 3 consecutive days of endurance training under hypoxia [inspired oxygen fraction (Fio_2_): 14.5%, HYP] or normoxia (Fio_2_: 20.9%, NOR). Each trial was separated by at least 2 weeks. During 3 consecutive days, subjects performed 90 min of endurance exercise session per day. Each training session consisted of high-intensity endurance interval exercise followed by 30 min of continuous exercise during 3 consecutive days (days 1–3). Before the training period (day 1) and during days 2–4 (day 4: the following morning after completing 3 consecutive days of training), all subjects arrived at the laboratory at 8:00 am following an overnight fast to determine baseline levels of muscle damage and inflammation. Body composition, fatigue and muscle soreness scores, muscular performance of the lower limb, bioimpedance (BI)-value, and muscle thickness of vastus lateralis were evaluated. Venous blood samples were also collected to determine serum CK, hsCRP, and free testosterone concentrations. After completing 3 consecutive days of endurance training, subjects performed an incremental exercise test (IET) to evaluate endurance performance, which reflects the magnitude of training stress over 3 consecutive days of endurance training.

### Training Protocols

During days 1–3, all exercise sessions were conducted using a cycle ergometer (Aerobike 75XLIII; Konami Corporation, Tokyo, Japan) in an environmentally controlled chamber. The hypoxic chamber used in this study was the whole-room type, and the hypoxic condition was established by insufflation of nitrogen. After a 5-min warm-up at 50 W, the subjects performed interval training (10 × 4-min pedaling at 80% of $\dot{\text{V}}{\text{o}}_{\text{2max}}$ with 2 min of active rest at 40% of $\dot{\text{V}}{\text{o}}_{\text{2max}}$ between sets) followed by 30-min continuous exercise at 60% of $\dot{\text{V}}{\text{o}}_{\text{2max}}$. The exercise intensity during the interval training session and continuous exercise session were relatively matched (%$\dot{\text{V}}{\text{o}}_{\text{2max}}$) between HYP and NOR. Similar exercise protocols have been used in a previous study (Sumi et al., 2018) to evaluate the acute responses of muscle damage and inflammation following a single session of endurance exercise under hypoxia. To prevent psychological influence, the subjects were not informed of whether the trial was conducted under normoxia or hypoxia.

### Dietary Controls

All subjects consumed a standard diet for Japanese adult males, which was provided during the 3 consecutive days of endurance training. The total calorie consumed was approximately 2,300 kcal/day. The relative macronutrient consumption was 64% carbohydrate, 21% fat, and 15% protein.

### Measurements

#### $\dot{\text{V}}{\text{o}}_{\text{2max}}$ (Preliminary Measurement)

The $\dot{\text{V}}{\text{o}}_{\text{2max}}$-test was conducted twice under normoxia (Fio_2_: 20.9%) and hypoxia (Fio_2_: 14.5%). The test began at 50 W, and the load was increased progressively by 30-W increments every 2 min until exhaustion [80 revolutions/min (rpm)]. During the test, expired gases were collected and analyzed using an automatic gas analyzer (AE300S; Minato Medical Science Co., Ltd., Tokyo, Japan). The respiratory data were averaged every 30 s. Heart rate (HR) was measured continuously during the test using a wireless HR monitor (Accurex Plus; Polar Electro Oy, Kempele, Finland). The order of the two repeated bouts of $\dot{\text{V}}{\text{o}}_{\text{2max}}$-tests under normoxia and hypoxia was randomized. These tests were performed a minimum of at least 3 days before the trials commenced.

#### Measurements in the Main Trials (Days 1–4)

##### Body Composition

Body mass, fat mass, and fat-free mass were evaluated using a multifrequency impedance technique (InBody 720; Biospace, Seoul, Korea). Using a range of frequencies from 1 kHz to 1 MHz, the InBody 720 accurately measured the amount of body water and body composition including fat, free fat, and skeletal muscle masses (Deurenberg et al., 1989; Demura et al., 2004). Subjects emptied their bladders prior to the measurements.

##### Physiological and Rating of Perceived Exertion Responses During Exercise Sessions

Percutaneous oxygen saturation (Spo_2_) was measured using a finger pulse oximeter (Smart Pulse; Fukuda Denshi, Tokyo, Japan) placed on the tip of the right forefinger. During the interval training session, the average values of the final 1 min of the last set (10 set) were calculated. During the 30-min continuous exercise session, the average values of the final 1 min of 30 min were determined. Heart rate was recorded at the same time points as the Spo_2_ measurements. The subjects reported rating of perceived exertion (RPE)–respiratory strain (RPE-R) and RPE–leg strain (RPE-L) at the end of the interval training session and continuous exercise session (Wilson and Jones, 1991). Blood lactate concentrations were evaluated immediately after the interval exercise session using a lactate analyzer (Lactate Pro; Arkray Co., Kyoto, Japan). The physiological variables and the RPE during 3 consecutive days of interval and continuous exercise sessions were averaged to compare between the two trials.

##### Blood Variables

Throughout days 1–4, fasting blood samples were collected following at least 15 min of rest. All blood samples for determinations of blood gas and electrolytes were collected using a 2.5-ml syringe containing heparin. Another 20-ml syringe was used to obtain serum samples. Serum samples were obtained after 10 min of centrifugation at 4°C (3,000 rpm), and the samples were stored at −80°C until analysis. From obtained blood samples, serum CK, hsCRP, and free testosterone concentrations were measured. The serum CK, hsCRP, and free testosterone concentrations were measured at a clinical laboratory (SRL Inc., Tokyo, Japan). The intra-assay coefficients of variability were 3.1, 1.5, and 2.8% for the serum CK, hsCRP, and free testosterone, respectively.

##### Muscle Swelling

The BI-value and muscle thickness were evaluated to determine exercise-induced muscle swelling. The BI-value was measured locally at the midpoint (50% of the distance between the greater trochanter and patellar tendon) of the thigh using a specially developed device (TLM-2000; Toray Engineering Co., Ltd., Shiga, Japan). The point was marked to ensure measurements at the same place. The device can evaluate local BI-value using surface electromyogram (EMG) during submaximal isometric muscle contraction. In short, EMG was imported into a specially developed device, and external resistance was subsequently added to EMG data to calculate the attenuation rate (Murai et al., 2014). The attenuation rate means local BI of the exercise muscles, which reflects muscle swelling (increase in water volume in muscle tissues). Before the measurement, the skin was pretreated to reduce resistance between the electrode and skin using skin pretreatment gel (Skinpure YZ0019; Nihon Koden Co., Tokyo, Japan). Subsequently, the subjects performed submaximal isometric muscle contraction for 15 s with a knee extension at a knee angle of 135° (full extension of the lower leg was defined as 0°) with a 5-kg weight on the ankle. The average value of the two measurements was defined as the BI-value. The thickness of the vastus lateralis muscle was measured at the same place of BI-value using an ultrasound system (IPC-1531; Aloka, Tokyo, Japan). The average value of three trials was adopted.

##### Muscular Performance of Lower Limb

Maximal voluntary contraction (MVC) of knee extension and drop jump (DJ) were performed to evaluate changes in power output for the lower limb muscles. The MVC with the knee positioned at 75° of extension was measured using an isokinetic dynamometer (Biodex System 4; SAKAI Medical Co., Tokyo, Japan). The subjects performed two repeated bouts of maximal knee extension for 5 s, and the highest value was selected for further analysis. A 60-s rest period was provided between bouts. Subjects performed DJ from a 60-cm box. After landing on a platform, the subject was instructed to perform a maximal vertical jump with minimal contact time. The DJ index was calculated from the jump height and contact time (jump height/contact time) using a sensing mat platform (multi jump tester; DKH Corp., Tokyo, Japan). The DJ-test was repeated twice with 1-min rest period between jumps, and the highest DJ index was used for analysis.

##### Subjective Feelings of Fatigue and Muscle Soreness

Subjective feelings of fatigue and leg muscle soreness were evaluated using a 10-cm visual analog scale, with 0 cm indicating no pain or fatigue and 10 cm indicating the worst pain and fatigue (Sumi et al., 2018).

##### Endurance Performance Response

After completing 3 consecutive days of endurance training, subjects performed IET to determine endurance performance response. The IET was conducted in normoxic condition in both trials. The test began at 50 W, and the load was increased progressively by 30-W increments every 3 min until volitional exhaustion. The pedaling workload at volitional exhaustion was defined as maximal aerobic power output (MPO). Blood lactate concentrations were evaluated immediately after exhaustion.

### Statistical Analyses

Data are expressed as the mean ± standard deviation. Two-way analysis of variance (ANOVA) with repeated measures was used to test the interaction (trial × time) and main effect (trial, time). When ANOVA revealed a significant interaction or main effect, the Tukey–Kramer test was performed as a *post-hoc* analysis to identify differences. Effect size was evaluated by partial η^2^ (η_*p*_^2^) for two-way ANOVA with repeated measures and Cohen *d* for a paired *t*-test. For all tests, *P* < 0.05 was considered statistically significant.

## Results

### $\dot{\text{V}}{\text{o}}_{\text{2max}}$ and Pedaling Workload

$\dot{\text{V}}{\text{o}}_{\text{2max}}$ was significantly lower in hypoxia (42.2 ± 3.8 ml /kg per minute) than in normoxia (51.8 ± 4.8 ml/kg per minute: *P* < 0.0001, *d* = 0.99). Consequently, the pedaling workloads during interval exercise (HYP: 166 ± 4 W, NOR: 194 ± 8 W; *P* < 0.0001, *d* = 0.99) and 30-min continuous exercise (HYP: 105 ± 4 W, NOR: 132 ± 6 W; *P* < 0.0001, *d* = 0.99) were significantly lower in the HYP than NOR trial (Table 1).

**Table 1:** $\dot{\text{V}}{\text{o}}_{\text{2max}}$ and pedaling workload.

	**HYP**	**NOR**
$\dot{\text{V}}{\text{o}}_{\text{2max}}$ (ml/kg per min)	42.2 ± 3.8	51.8 ± 4.8
Interval exercise (W)	166 ± 4^†^	194 ± 8
Continuous exercise (W)	105 ± 4^†^	132 ± 6

*Values are mean ± SD. $\dot{\text{V}}{\text{o}}_{\text{2max}}$, maximal oxygen uptake*.

† *Significant difference vs. NOR*.

### Body Composition

Body weight significantly decreased on day 3 (NOR: 60.9 ± 5.7 kg, HYP: 60.8 ± 5.6 kg) and day 4 (NOR: 60.8 ± 5.6 kg, HYP: 60.6 ± 5.4 kg) compared with day 1 (NOR: 61.5 ± 5.9 kg, HYP: 61.4 ± 5.9 kg) in both trials (main effect for time: *P* = 0.002, η_*p*_^2^ = 0.447). However, there was no significant difference between the two trials. Body fat mass and fat-free mass did not significantly change throughout days 1–4 in both trials.

### Physiological and RPE Responses During Endurance Training

The average Spo_2_ during interval and continuous exercise was significantly lower in the HYP trial (85 ± 1.7%) than in the NOR trial (97 ± 0.5%; *P* < 0.0001, *d* = 0.99). By contrast, HR [HYP: 169 ± 9 beats/min (bpm), NOR: 171 ± 8 bpm], RPE-R (HYP: 6 ± 1, NOR: 6 ± 2), and RPE-L (HYP: 8 ± 1, NOR: 9 ± 1) did not significantly differ between the HYP and NOR trials. The blood lactate concentration immediately after the interval exercise was slightly higher in the HYP trial (7.8 ± 2.3 mmol/L) than in the NOR trial (6.0 ± 2.2 mmol/L; *P* = 0.10).

### Blood Variables

Table 2 presents the changes in blood variables. Serum CK and hsCRP concentrations did not significantly change during days 1–4 in both trials. Moreover, there was no significant difference between the two trials at any time points. Serum free testosterone concentrations significantly decreased on day 4 compared with day 1 in NOR (main effect for time: *P* = 0.023, η_*p*_^2^ = 0.339). However, HYP showed no significant change in serum-free testosterone concentrations on days 1–4. Furthermore, no significant difference was observed in serum-free testosterone concentrations between the two trials.

**Table 2:** Changes in blood variables.

		**Day 1**	**Day 2**	**Day 3**	**Day 4**	**Time × trial**	**Main effect**	
							**Time**	**Trial**
CK (U/L)	HYP	388 ± 330	306 ± 171	319 ± 253	335 ± 350	*P* = 0.752	*P* = 0.914	*P* = 0.114
	NOR	218 ± 130	237 ± 111	211 ± 96	194 ± 87			
hsCRP (ng/ml)	HYP	238 ± 200	222 ± 142	181 ± 145	162 ± 185	*P* = 0.515	*P* = 0.269	*P* = 0.536
	NOR	476 ± 815	317 ± 323	234 ± 167	180 ± 133			
Free testosterone (pg/ml)	HYP	23.3 ± 6.7	22.8 ± 4.9	20.0 ± 7.8	20.3 ± 3.8	*P* = 0.126	*P* = 0.023	*P* = 0.497
	NOR	23.6 ± 7.0	20.0 ± 7.9	20.2 ± 4.7	17.5 ± 5.7^*^			

*Values are mean ± SD. CK, creatine kinase; hsCRP, high-sensitivity C-reactive protein*.

* *Significant difference vs. day 1*.

### Muscle Swelling

Table 3 shows changes in muscle thickness and BI-value. The muscle thickness significantly increased on day 4 compared with day 1 in NOR (main effect for interaction: *P* = 0.014, η_*p*_^2^ = 0.373). However, HYP showed no significant change in muscle thickness during days 1–4. Moreover, there was no significant difference between the two trials at any time points. The BI-value significantly decreased with training in both trials (main effect for time: *P* < 0.0001, η_*p*_^2^ = 0.558), whereas no significant difference was observed between the two trials.

**Table 3:** Changes in MVC, DJ index, and muscle swelling variables of lower limb.

		**Day 1**	**Day 2**	**Day 3**	**Day 4**	**Time × trial**	**Main effect**	
							**Time**	**Trial**
MVC (%)	HYP	100 ± 0	98 ± 5	92 ± 6^*^	95 ± 9	*P* = 0.198	*P* < 0.0001	*P* = 0.085
	NOR	100 ± 0	91 ± 6^*^	90 ± 7^*^	91 ± 7^*^			
DJ index (%)	HYP	100 ± 0	95 ± 13	88 ± 13^*^	92 ± 12	*P* = 0.575	*P* = 0.008	*P* = 0.535
	NOR	100 ± 0	90 ± 10	87 ± 15^*^	92 ± 11			
Muscle thickness (cm)	HYP	4.31 ± 0.3	4.33 ± 0.4	4.33 ± 0.4	4.32 ± 0.5	*P* = 0.014	*P* = 0.396	*P* = 0.270
	NOR	4.21 ± 0.5	4.22 ± 0.4	4.29 ± 0.4	4.34 ± 0.4^*^			
BI (kΩ)	HYP	14.6 ± 6.9	12.4 ± 5.0	11.5 ± 4.3	9.0 ± 4.0^*^	*P* = 0.740	*P* < 0.0001	*P* = 0.499
	NOR	15.5 ± 6.8	12.9 ± 5.8	12.1 ± 6.7^*^	11.7 ± 6.7^*^			

*Values are mean ± SD. MVC, maximal voluntary contraction; DJ index, drop jump index; BI, bioimpedance*.

* *Significant difference vs. day 1*.

### Muscular Performance of the Lower Limb

Table 3 presents changes in the MVC and DJ index. MVC (main effect for time: *P* < 0.0001, η_*p*_^2^ = 0.580) and DJ index (main effect for time: *P* = 0.008, η_*p*_^2^ = 0.425) significantly decreased with training in both trials. However, there was no significant difference in both MVC and DJ index between the two trials.

### Subjective Feelings of Muscle Soreness and Fatigue

Table 4 shows changes in subjective feelings of muscle soreness and fatigue. In the NOR trial, the subjective feeling of muscle soreness increased significantly during days 2–4 compared with day 1 (main effect for time: *P* = 0.002, η_*p*_^2^ = 0.618). Moreover, the HYP trial showed no significant change in the subjective feelings of muscle soreness during days 1–4. However, there was no significant difference between the two trials. The subjective feelings of fatigue significantly increased with training in both trials (main effect for time: *P* = 0.003, η_*p*_^2^ = 0.617). Furthermore, the HYP trial showed a significantly lower score of fatigue on day 4 compared with the NOR trial (main effect for trial: *P* = 0.004, η_*p*_^2^ = 0.672).

**Table 4:** Changes in subjective feelings of fatigue and muscle soreness.

		**Day 1**	**Day 2**	**Day 3**	**Day 4**	**Time × Trial**	**Main effect**	
							**Time**	**Trial**
Muscle soreness (cm)	HYP	2.1 ± 3.0	2.3 ± 2.0	4.3 ± 2.6	4.1 ± 2.4	*P* = 0.150	*P* = 0.002	*P* = 0.439
	NOR	1.1 ± 1.1	3.1 ± 2.4^*^	4.9 ± 2.4^*^	4.4 ± 1.8^*^			
Fatigue (cm)	HYP	1.9 ± 2.8	2.6 ± 1.3	5.6 ± 2.3^*^	5.5 ± 2.4^*^^†^	*P* = 0.146	*P* = 0.003	*P* = 0.004
	NOR	1.5 ± 1.7	3.9 ± 2.5^*^	6.5 ± 2.3^*^	6.7 ± 2.3^*^			

*Values are mean ± SD*.

* *Significant difference vs. day 1*.

† *Significant difference vs. NOR*.

### Endurance Performance

On day 4, there was no significant difference in MPO between the two trials (NOR: 290 ± 28 W, HYP: 297 ± 27 W). Furthermore, blood lactate concentrations immediately after exhaustion (NOR: 13.2 ± 3.4 mmol/L, HYP: 12.5 ± 3.1 mmol/L) did not significantly differ between the two trials.

## Discussion

In this study, we determined the effects of 3 consecutive days of endurance training under hypoxia on muscle damage and inflammatory responses. A novel finding was that 3 consecutive days of endurance training under hypoxia induced a lower score of subjective feelings of fatigue. However, changes in blood variables (serum CK and hsCRP concentrations), muscle swelling (BI-value and muscle thickness), and muscular performance (MVC and DJ index) did not significantly differ compared with those following the same training under normoxia. Furthermore, endurance performance response after completing 3 consecutive days of endurance training was comparable between the two trials.

The score of subjective feeling of fatigue was significantly lower in the HYP trial than in the NOR trial on day 4. In the present study, exercise intensity for pedaling exercise was relatively matched (% of $\dot{\text{V}}{\text{o}}_{\text{2max}}$ evaluated under each environment) between the HYP and NOR trials. Because of the significantly lowered $\dot{\text{V}}{\text{o}}_{\text{2max}}$ in hypoxia, the HYP trial used significantly lower absolute exercise intensity (i.e., pedaling workload) compared with the NOR trial (interval exercise, HYP: 166 ± 4 W, NOR: 194 ± 8 W). Our previous study established that a single bout of prolonged running under hypoxia caused lower exercise-induced serum Mb elevations compared with the same equivalent exercise (same % of $\dot{\text{V}}{\text{o}}_{\text{2max}}$ in normoxia and hypoxia) under normoxia (Sumi et al., 2018). We speculated that lower mechanical stress (i.e., running velocity) in hypoxia (average running velocity: 16.2 ± 0.3 km/h) would be responsible for lower exercise-induced muscle damage compared with the exercise under normoxia (average running velocity: 18.0 ± 0.3 km/h). Therefore, the lower fatigue score in the HYP trial would be mainly explained by lower mechanical stress (i.e., lower pedaling workload) during daily 90-min endurance exercise session. In contrast, endurance performance response in the following morning after completing 3 consecutive days of endurance training (day 4) did not significantly differ between the two trials. Because no significant difference was observed in muscle damage and inflammatory responses between the HYP and NOR trials evaluated by blood variables, muscle swelling, and muscular performance, the lack of a difference in endurance performance response was not surprising. Hypoxic training has been widely accepted as a potent tool for improving endurance capacity among endurance athletes, and a large amount of experimental evidence supports the efficacy of this training procedure (Dufour et al., 2006; Czuba et al., 2011, 2017). Based on the present findings, hypoxic training can be expected to improve endurance capacity with lower subjective feeling of fatigue. Furthermore, as muscle damage and inflammatory markers (e.g., serum CK, hsCRP concentrations) did not differ significantly between HYP and NOR trials, hypoxic training would not increase the risk of overtraining syndrome and injury compared with the same training under normoxia.

In the present study, we selected pedaling exercise during 3 consecutive days of endurance training. Pedaling exercise includes lower eccentric muscle contraction compared with running exercise (Nieman et al., 2014; Mallol et al., 2020). Indeed, muscle damage (serum CK) and inflammatory (serum hsCRP) markers in blood did not change with 3 consecutive days of endurance training in both trials. In study contrast, a previous demonstrated that 3 consecutive days of running exercise (75 min/day) increased baseline serum Mb, CK, and hsCRP concentrations (Ishibashi et al., 2017). Moreover, Sim et al. (2014) established that 7 consecutive days of pedaling exercise did not increase baseline serum CRP concentrations, whereas the same duration of training using running presented higher concentrations. Therefore, muscle damage and inflammatory responses following endurance training may differ according to the exercise modality used (e.g., running vs. pedaling). However, increased score of subjective feeling (muscle soreness and fatigue) and reduction of muscular performance occurred following endurance training in both the HYP and NOR trials. As the accumulation of metabolite in working muscle promotes muscle swelling and reduction of muscular performance even if eccentric muscle contraction is not mainly recruited (Brandner and Warmington, 2017; Alvarez et al., 2020), it appears that increased score of subjective feeling and reduction of muscular performance were observed following pedaling training.

As another hypoxic training procedure, exercise training with blood flow restriction (BFR) has been known to improve endurance capacity and muscle function (Fahs et al., 2014, 2015). Exercise with BFR typically decreases oxygen saturation in working muscle [i.e., lower tissue oxygen saturation (Sto_2_)], which leads to augmented local hypoxia (Karabulut et al., 2014). In contrast, exercise under normobaric hypoxia (systemic hypoxia) lowers both Spo_2_ and Sto_2_ (Van Thienen and Hespel, 2016). Therefore, physiological responses would be different between exercise under normobaric hypoxia (systemic hypoxia) and exercise with BFR (local hypoxia), although both training procedures are included in “hypoxic training” (Girard et al., 2017). Thus, further studies are needed to compare muscle damage and inflammatory responses between endurance training under normobaric hypoxia and endurance training with BFR.

The present study had several limitations. We selected pedaling exercise during the training period. Exercise involving an eccentric contraction promotes exercise-induced muscle damage (Bruunsgaard et al., 1997; Child et al., 1998; Proske and Allen, 2005). Because pedaling exercise contains lower eccentric muscle contraction, the magnitude of muscle damage and inflammatory responses would be lower compared with running exercise. Therefore, the determination of impact of different types of exercise training (e.g., running or resistance exercise) under hypoxia would be meaningful. Moreover, the findings from this study may be specific to relatively short-term (3 consecutive days) endurance training. We especially focused on short-term (3 days) endurance training to assess the influence of rapid increases in training-induced stress. This strategy was chosen as an attempt to mimic transient increases in training volume, which are frequently seen under real situation during training program (e.g., during a training camp). In addition, the same endurance training strategy was previously shown to increase muscle damage and inflammatory response (Ishibashi et al., 2017). However, further studies are needed to confirm whether our findings can be applied to long-term training regimens. Finally, we were not able to set another trial using the same absolute exercise intensity between hypoxia and normoxia. In the previous study, endurance exercise under hypoxia caused greater IL-6 elevation compared with exercise at the same absolute exercise intensity (i.e., same running velocity) under normoxia (Hill et al., 2020). Therefore, muscle damage and inflammatory responses after endurance training hypoxia may be augmented when the same absolute exercise intensity is selected under normoxia.

## Conclusion

Three consecutive days of endurance training under hypoxia induced similar levels of muscle damage, inflammation, and performance responses compared with the same training in normoxia. This finding suggests that several days of endurance training under normobaric hypoxia does not aggravate muscle damage, inflammation, and reduction of endurance performance. Therefore, endurance training under normobaric hypoxia can be used to improve endurance capacity (Dufour et al., 2006; Czuba et al., 2011, 2017) without increasing the risk of overtraining or decreasing the physical condition compared with the same training under normoxia, as long as the exercise intensity is relatively matched.

## Data Availability Statement

The datasets generated for this study are available on request to the corresponding author.

## Ethics Statement

The studies involving human participants were reviewed and approved by This study was approved by the Ethics Committee for Human Experiments at Ritsumeikan University (BKC-IRB-2018-093), and was conducted in accordance with the Declaration of Helsinki. The patients/participants provided their written informed consent to participate in this study. Written informed consent was obtained from the individual(s) for the publication of any potentially identifiable images or data included in this article.

## Author Contributions

DS and KG contributed to the study design, data collection, analysis, and manuscript writing. KY contributed to the study design and data collection. All authors contributed to the article and approved the submitted version.

## Conflict of Interest

The authors declare that the research was conducted in the absence of any commercial or financial relationships that could be construed as a potential conflict of interest.
